# Resveratrol inhibits migration and Rac1 activation in EGF- but not PDGF-activated vascular smooth muscle cells

**DOI:** 10.1002/mnfr.201100309

**Published:** 2011-07-05

**Authors:** Mario Kumerz, Elke H Heiss, Daniel Schachner, Atanas G Atanasov, Verena M Dirsch

**Affiliations:** Department of Pharmacognosy, University of ViennaVienna, Austria

**Keywords:** Lamellipodia, Migration, Rac1, Resveratrol, Vascular smooth muscle cells

## Abstract

**Abstract:**

**Scope:** Migration of vascular smooth muscle cells (VSMC) reflects one of the initial steps in atherosclerosis. Resveratrol (RV) is suggested to mediate putative vasoprotective properties of red wine leading to the hypothesis that RV interferes with growth factor-induced migration of VSMC.

**Methods and results:** We show here that RV (50 μM) strongly reduces epidermal growth factor (EGF)- but not platelet-derived growth factor (PDGF)-induced VSMC migration using the wound-healing technique. Accordingly, RV inhibited Rac1 activation and lamellipodia formation in response to EGF but not PDGF as shown by pull-down assays and fluorescence microscopy after actin staining with phalloidin-FITC, respectively. Since Src-family kinases and the phosphatidylinositol-3 kinase (PI3K) are reported to be crucial upstream mediators of Rac1 activation we examined the PI3K inhibitor wortmannin and the src kinase inhibitor SU6656 side-by-side with RV for their anti-migratory potential. Whereas src inhibition abrogated both EGF- and PDGF-triggered migration, wortmannin, like RV, was more effective in EGF- than PDGF-activated cells, suggesting that PI3K inhibition, previously shown for RV in growth factor-activated VSMC, contributes to the anti-migratory effect of RV in EGF-stimulated VSMC.

**Conclusion:** This study is the first to discover an anti-migratory potential of RV in EGF-activated VSMC that is most likely mediated via Rac1 inhibition.

## 1 Introduction

The polyphenolic compound resveratrol (RV) is a phytoalexin produced by certain plants in response to injury, stress, UV light or infection, which is predominantly found in berries, nuts and grapes [1]. RV is discussed to play a major role in the French paradox, the low risk to develop cardiovascular diseases in France despite a diet rich in saturated fatty acids. In the last decade, great efforts were made to scientifically prove the health-beneficial effects of RV, and several molecular targets have been unravelled involved in inflammation, migration or proliferation [2, 3].

Atherosclerosis, blood vessel narrowing in response to inflammation and lipid accumulation, is a multi-step process and involves diverse subtypes of cells and tissues [4]. Vascular smooth muscle cells (VSMC) play a crucial role in many stages of atherosclerosis [4, 5], including growth factor-triggered migration of VSMC into the intima of the vessel and subsequent initiation of proliferation which gives rise to the progression of the disease [6]. Platelet-derived growth factor (PDGF) is the most important pro-migratory stimulus for VSMC [6, 7]. Most interestingly, angiotensin II, also an important growth factor in atherogenesis was recently reported to induce VSMC migration via the transactivation of the EGF-receptor [8]. In addition, EGF and related proteins (e.g. HB-EGF, TGFα) are expressed by cells involved in atherogenesis and appear to mediate important biological effects related to this process [9]. EGF and cognate molecules such as HB-EGF are reported directly or indirectly to act as mito- and motogens in VSMC [6, 7]. At the molecular level, migration is orchestrated by several key regulators, including the small GTPases RhoA, cdc42 and Rac1, and several stimuli have been demonstrated to activate GTPases in VSMC, among others PDGF and EGF [6, 10].

Since RV has been documented to inhibit migration in cancer cells [11] and VSMC migration is an initial step in the progression of atherosclerosis, we aimed to investigate a possible inhibitory role of RV on VSMC migration in response to two important stimuli, PDGF and EGF.

## 2 Materials and methods

### 2.1 Reagents

RV, phalloidin-FITC, wortmannin and SU6656 were purchased from Sigma Aldrich (St. Louis, MO, USA). EGF was bought from Millipore (Temecula, CA, USA) and PDGF-BB was purchased from Bachem (Weil am Rhein, Germany). Rac1 and cdc42 activation assay kits including PAK-PBD agarose beads and Western Blot antibodies targeting Rac1 and cdc42 were bought from Cell Biolabs (San Diego, CA, USA).

### 2.2 Cell culture

Rat VSMC were isolated from thoracic aortas of male Sprague–Dawley rats by enzymatic digestion as described elsewhere [12] and VSMC between passages 7 and 15 were used for all experiments. Cells were cultured in Dulbecco's Modified Eagles Medium (DMEM, Lonza, Basel, Switzerland) containing 10% calf serum (CS), antibiotics and l-glutamine at 37°C and 5% CO_2_. Before stimulation, VSMC were serum-starved by incubation with DMEM containing 0.1% CS, antibiotics and l-glutamine for 24–48 h.

### 2.3 Cell migration (wound-healing technique)

For the quantification of cell migration, VSMC were grown in 6-well plates to 95% confluence and serum-starved for 24 h. For each well, two scratches were made using a sterile 100–1000 μL tip and detached cells were washed away. Thereafter, starving of VSMC was continued for additional 24 h. Before induction of migration, four distinct and accurately defined scratch areas per well (each in duplicate) were photographed with 200-fold magnification. Nearly 21 h after stimulation, pictures of the same regions were taken and paired images were analyzed for the repopulation of the scratched areas (cell profiler software, Broad Institute Imaging Platform).

### 2.4 Fluorescence staining

To visualize the actin cytoskeleton, 5×10^4^ cells were seeded on coverslips placed in 12-well plates. VSMC were serum-starved for 24 h and then treated with different stimuli. After washing with PBS, cells were fixed on coverslips as described [13]. Samples were washed thrice with PBS and incubated with a 1:200 dilution of phalloidin-FITC (in PBS) for 30 min at room temperature in dark. Plates were rinsed thrice with PBS before mounting on glass slides. Samples were dried at room temperature and then viewed with a fluorescence microscope (BX51 microscope, Olympus, Hamburg, Germany).

### 2.5 GTPase activity assay

PAK-PBD agarose beads which specifically bind GTP-coupled active Rac1 and cdc42 were used as recommended by the supplier. VSMC were seeded in 10 cm dishes and grown to 90% confluence. After 48 h of serum starvation cells were stimulated, washed twice with ice-cold PBS and subsequently lysed. Before the pull-down, crude cell lysates were pre-cleared and insoluble material was pelleted for 4 min at 4°C. In total, 20 μL of the lysate was taken away for the loading control before the remains (800 μg protein/sample) was mixed with 20 μL of bead slurry. Samples were incubated for 45 min at 4°C in a rotator and then washed thrice with cell lysis buffer. Beads were mixed with 15 μL of 3× SDS-PAGE Dye and heated for 5 min at 95°C before loading on 15% PAA gels. Obtained signals of pulled-down GTPases were normalized to the loading controls of the respective lysates.

### 2.6 Statistics

Data were evaluated using the GraphPad PRISM (version 4.03) and are expressed as mean±SEM. If not otherwise stated, two-tailed paired *t*-test was applied and *p*<0.05 was considered significant.

## 3 Results

### 3.1 PDGF but also EGF induces migration of VSMC

**Figure 1:**
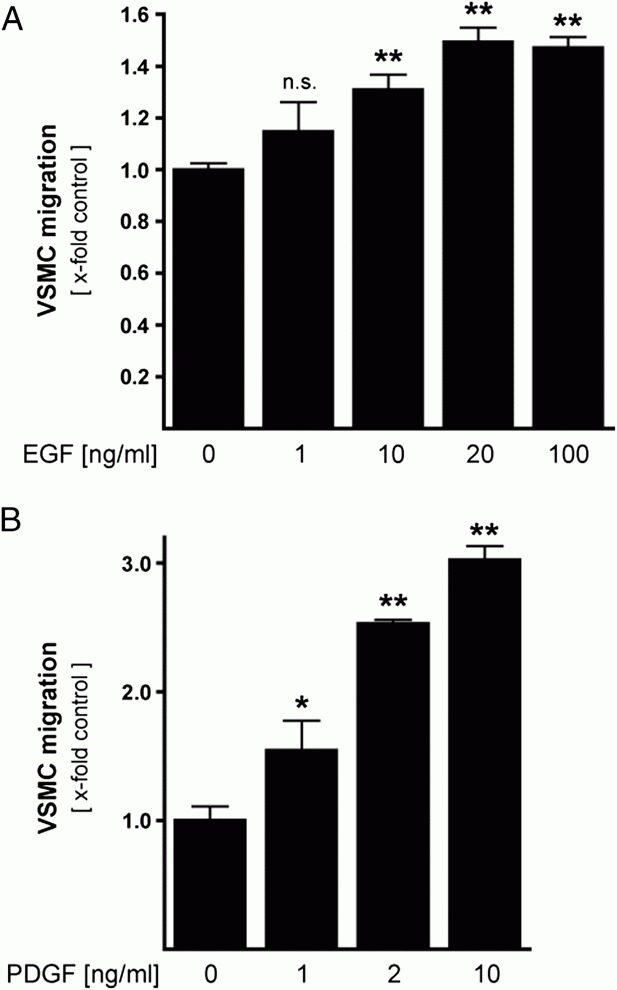
EGF and PDGF induce migration of VSMC in a dose-dependent manner. VSMC were exposed to increasing concentrations of (A) EGF (1–100 ng/mL) or (B) PDGF (1–10 ng/mL). Migration was determined by the wound-healing assay as described in Section 2. Pictures were taken before and after 21 h of stimulation with the indicated concentrations of EGF or PDGF and then evaluated. Bar graphs show the mean±SEM of three independent experiments (^*^p<0.05; ^**^p<0.01; ns, not significant; one-way ANOVA).

Compared with PDGF, EGF is less well documented as migratory stimulus in VSMC. Therefore and in order to determine the optimal dose for our studies, we first conducted wound-healing experiments using different concentrations of EGF between 1 and 100 ng/mL. While migration induced by 1 ng/mL EGF did not reach significance, 20 ng/mL EGF led to a 50% increase in VSMC migration, which could not be further enhanced by applying higher concentrations of EGF (Fig. 1A). Thus, 20 ng/mL EGF was used for all subsequent experiments to initiate migration. To verify that the observed effect was due to migration but not proliferation, VSMC were pretreated with mitomycin C, a potent inhibitor of cell proliferation, prior to stimulation with EGF (20 ng/mL). Mitomycin C did not alter the capacity of EGF to induce closure of the scratch (data not shown) underpinning the migratory stimulus by EGF. PDGF, which is reported to be a stronger migratory stimulus than EGF [6], dose-dependently induced migration also in our hands at concentrations of 1–10 ng/mL (Fig. 1B).

### 3.2 RV preferentially abrogates EGF-induced migration

**Figure 2:**
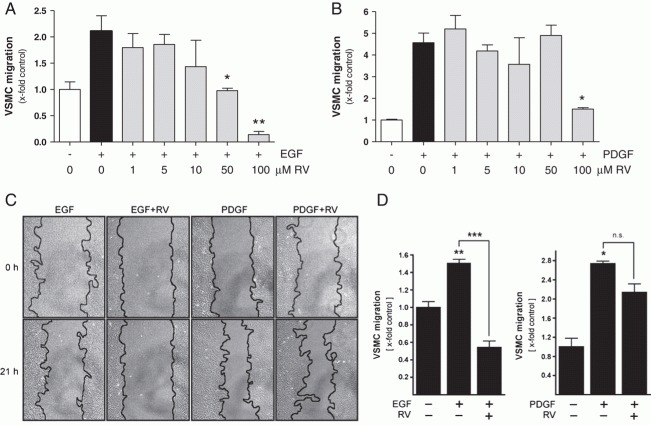
RV preferentially blocks EGF-induced migration. VSMC were preincubated with different concentrations of RV or vehicle (DMSO) for 30 min, and then EGF (20 ng/mL) (A) or PDGF (2 ng/mL) (B) were added for 21 h. Migration was assessed and quantified as described in Section 2. Bar graphs represent fold migration of unstimulated control and depict mean±SEM of three independent experiments (^*^p<0.05; ^**^p<0.01; ANOVA, Dunnett's post test versus stimulated control (black bar)). (C) VSMC were preincubated with RV (50 μM) or vehicle (DMSO) for 30 min and then EGF (20 ng/mL) or PDGF (2 ng/mL) was added for 21 h. Representative pictures taken from the wound-healing assay before and after growth factor stimulation. (D) Quantitative evaluation of wound healing assays. VSMC were treated as explained in (C). Bar graphs show the mean±SEM of at least three independent experiments. Arbitrary units were normalized to DMSO-treated cells and are expressed as ×-fold control (^*^p<0.05; ^**^p<0.01; ^***^p<0.001; ns, not significant).

Next we examined whether RV is able to influence EGF- or PDGF-induced migration. Wound-healing experiments were performed using 20 ng/mL EGF and 2 ng/mL PDGF, respectively, as well as different concentrations of RV ranging from 1 to 100 μM. These concentrations did not negatively affect viability of VSMC as assessed by Trypan Blue exclusion (Supplementary Information Fig. 1). As seen in Fig. 2], RV dose-dependently impairs EGF-triggered migration whereas PDGF-stimulated migration is only affected by 100 μM RV. EGF-induced migration is already slightly impaired by 10 μM RV and markedly suppressed by 50 μM RV (Fig. 2A and B). In order to better visualize and confirm the selectivity of RV towards EGF- over PDGF-mediated migration, we repeated the experiments with 50 μM RV, the concentration aimed to be used for all further investigations. Figure 2C shows representative pictures before and after an incubation period of 21 h with the different stimuli applied. Whereas 50 μM RV was able to completely block EGF-induced migration, it only slightly reduced PDGF-triggered migration. Figure 2D, depicting compiled data of at least three independent wound-healing experiments, shows that 50 μM RV reduced EGF-mediated migration by 67%, whereas the observed decrease in PDGF-triggered VSMC did not reach significance. Moreover, RV was still able to block EGF- but not PDGF-induced migration, even when higher concentrations of the respective growth factors were used (data not shown).

### 3.3 RV reduces lamellipodia formation

**Figure 3:**
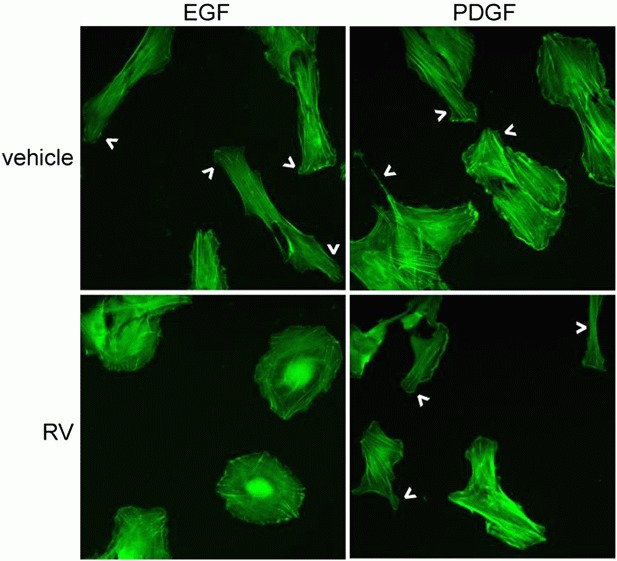
RV reduces lamellipodia formation of EGF- but not PDGF-activated VSMC. RV-preincubated VSMC (30 min) were stimulated with EGF (20 ng/mL) or PDGF (2 ng/mL) for 2 h. After washing and fixation, cells were stained with FITC-conjugated phalloidin for 30 min to visualize substructures of the actin cytoskeleton. Dried samples were monitored using a fluorescence microscope. Arrows indicate developed lamellipodia.

To unravel a putative influence of RV on actin substructures, we employed cytoskeleton staining of VSMC using phalloidin-FITC. Figure 3 shows representative photographs of phalloidin-stained cells after 2 h of EGF or PDGF stimulation (20 and 2 ng/mL, respectively) with or without RV pretreatment (50 μM, 30 min). In both, EGF- and PDGF-activated cells formation of lamellipodia (indicated with arrows) and stress fibres are visible indicating the induction of cell migration [14]. Pretreatment with RV abrogated lamellipodia formation in EGF- but not in PDGF-stimulated VSMC, obvious by the round cell shape in the case of EGF-treated cells. Moreover, addition of RV had no marked effect on stress fibre formation, neither in EGF- nor PDGF-stimulated VSMC.

### 3.4 EGF-induced Rac1 activation is abolished by RV treatment

**Figure 4:**
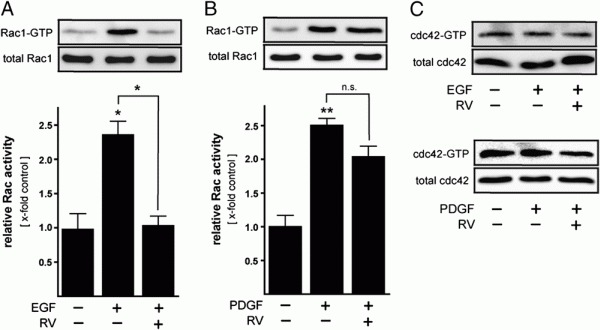
EGF- but not PDGF-activated Rac1 is inhibited by RV; cdc42 remains unaffected. VSMC were pretreated with RV or vehicle control (DMSO) for 30 min and then stimulated with 20 ng/mL EGF (A, C) or 2 ng/mL PDGF (B, C) as indicated for 5 min (for Rac1) and 2 min (for cdc42). Cells were subsequently lysed, and an aliquot was used to determine total Rac1 or total cdc42. The remaining lysate was incubated with PAK-PBD agarose beads for 45 min. Active pulled-down Rac1 was correlated to total Rac1 levels. Bar graphs show the mean±SEM of four independent experiments (^*^p<0.05; ^**^p<0.01; ns, not significant). Representative Western Blots are shown.

The major molecular requirement for lamellipodia formation is activation of the small GTPase Rac1 [14]. Figure 4A shows, that 20 ng/mL EGF caused a 2.4-fold Rac1 activation after 5 min that was completely inhibited by preincubation with 50 μM RV for 30 min. PDGF at 2 ng/mL induced a comparable Rac1 activation (2.5-fold), which was almost unaffected by RV (Fig. 4B). On the other hand cdc42 activity, which is supposed to be responsible for filopodia development was affected neither by EGF nor PDGF. Moreover, preincubation with RV had no effect on cdc42 activity (Fig. 4C). In accordance, no apparent differences in filopodia formation could be observed upon examination of the cell morphology after phalloidin-FITC staining (Fig. 3] and data not shown). Owing to unchanged stress fibre formation after treatment with RV an involvement of RhoA in this setting was considered unlikely.

### 3.5 The PI3K inhibitor wortmannin leads to a similar inhibition pattern than RV in EGF- versus PDGF-activated VSMC

**Figure 5:**
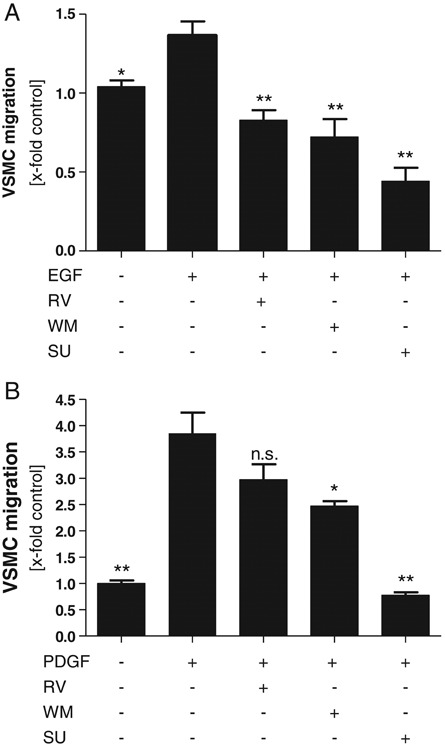
Inhibition of PI3K reduces cell migration more effectively in EGF- than in PDGF-activated VSMC. (A) VSMC were preincubated with vehicle (DMSO), RV, or the PI3K inhibitor wortmannin (WM, 50 nM), or the src inhibitor SU6656 (SU, 2 μM) for 30 min and then EGF (20 ng/mL) (A) or PDGF (2 ng/mL) (B) was added for 21 h. Migration of VSMC was determined by the wound-healing assay as described in Section 2 after 21 h of stimulation with the indicated compounds. Bar graphs show the mean±SEM of at least three independent experiments. Arbitrary units were normalized to DMSO-treated cells and are expressed as ×-fold control (^*^p<0.05; ^**^p<0.01; ns, not significant, ANOVA).

Almost nothing is reported about the regulation of Rac1 activation in EGF-activated VSMC. In other cell types, however, Rac1 activation seems to depend largely on the activation of PI3K and src family kinases [15]. We, therefore, examined the PI3K inhibitor wortmannin (50 nM) and the src inhibitor SU6656 (2 μM) side-by-side with RV regarding their inhibitory potential towards EGF- or PDGF-induced migration. Figure 5A shows that RV and wortmannin inhibited EGF-induced VSMC migration equally well below control levels; the src inhibitor was even more effective, altogether indicating that PI3K and src family kinases are both required to mediate EGF-induced migration in VSMC. In contrast, Fig. 5B shows that PI3K inhibition via wortmannin is much less effective (about 45% inhibition) in blocking PDGF-induced migration than src kinase inhibition (100%). The inhibitory potential of RV was visible but did not reach significance (see also Fig. 2C). Overall, in both settings, the inhibition pattern of RV resembled more the pattern of wortmannin than that of SU6656, which is in accordance with previous findings demonstrating that RV (50 μM) is able to block recruitment of the p85 subunit of PI3K to the adapter protein Gab1 in EGF-activated VSMC and thus interfering with the PI3K/Akt signalling pathway [16].

## 4 Discussion

Here we show, to the best of our knowledge, for the first time that RV is able to inhibit EGF-induced migration of VSMC, which is of considerable importance since angiotensin II-induced migration was shown to be routed via the EGF receptor and angiotensin II is a strong pro-atherogenic factor [8]. Furthermore, 15(S)-HETE was shown to lead to the transactivation of the EGF receptor with subsequent Src-Jak2-STAT3-dependent MCP-1 expression leading to vascular wall remodeling [17].

Most interestingly, RV did not readily block PDGF- but EGF-induced migration of VSMC. This effect was not due to the different concentrations of growth factors used, since very similar inhibition patterns were found when using higher amounts of either growth factor. In fact, a very recent study confirms our result: The group around H. J. Lee showed that piceatannol but not RV (both at 20 μM) strongly inhibited PDGF-induced migration of human aortic smooth muscle cells [18]. The finding that an anti-migratory effect of RV in VSMC may be stimulus-dependent is also supported by Venkatesan et al. They report that RV is able to inhibit IL-18 and extracellular matrix metalloproteinase inducer (EMMPRIN, CD147)-induced smooth muscle cell migration [19].

Phalloidin staining of EGF-activated VSMC revealed that RV mainly affected lamellipodia formation. In order to confirm these microscopically observed changes in the actin architecture on the molecular level we conducted pull-down assays targeting GTP-bound small GTPases, which are central players for the regulation of actin remodelling. The growth factor-induced kinetics of GTPase activation is cell-type specific and furthermore varies among the distinct GTPases. Whereas cdc42 and Rac1 become activated within minutes [10], RhoA is often shown to bind GTP after 30–90 min [20, 21]. We could clearly show that impaired lamellipodia formation is indeed associated with inhibition of Rac1 activation in EGF- but not in PDGF-stimulated VSMC after 5 min. These observations go along with the widely accepted model, that Rac1 is mainly responsible for the constitution of lamellipodia [14]. Since PDGF-activated Rac1 was not significantly inhibited by RV, it appears unlikely that RV acts as an immediate inhibitor of Rac1-associated guanine nucleotide exchange factor (GEF) or as GTPase activating protein (GAP) activator, but rather further upstream of the GTPase. Important upstream signalling molecules in EGF-mediated migration seem to be PI3K and the src family kinases [7, 14, 15]. We therefore examined respective inhibitors side-by-side with RV to compare their anti-migratory activity in EGF- versus PDGF-activated VSMC. Inhibition of src blocked VSMC migration in response to both stimuli. The PI3K inhibitor wortmannin, however, was less effective in PDGF- than in EGF-activated VSMC, resembling RV in its selective inhibition of EGF-triggered migration (Fig. 2 and 5). This suggests – although not proves – that RV may act via inhibition of the PI3K signalling pathway. In fact, we have shown previously that RV inhibits PI3K p85 phosphorylation and membrane recruitment in angiotensin II- or EGF-activated VSMC downstream of the EGF receptor whereas src kinase was excluded as target for RV [16, 22]. Interestingly, Iijima et al. [23] showed that a red wine polyphenol fraction inhibited VSMC migration via two distinct mechanisms, namely via inhibition of PI3K and the p38 signalling pathway. Since we and others showed that RV does not affect growth factor-induced p38 activation [18, 22, 24], RV may contribute to the first but not the latter mechanism of the examined red wine polyphenol extract [23]. As polyphenol and radical scavenger RV may be suspected to mediate its inhibitory effects on EGF-mediated migration by antioxidant mechanisms. However, our data show that a redox-inactive derivative of RV, *trans*-3,5-dihydroxy-4′-methoxystilbene [24], is still able to inhibit EGF-mediated migration (Supplementary Information Fig. 2). These findings are in line with our observation that inhibition of EGF-induced Akt phosphorylation is redox-independent [24].

Filopodia formation was hardly detected microscopically and changes in cdc42 activation, supposed to significantly contribute to filopodia formation [25], were not observed upon EGF, PDGF or RV stimulation. Although we were able to detect single filopodia under the microscope, their capillary and needle-like structure made it difficult to precisely quantify the number per cell or their structure. At the molecular level, cdc42 activity was evaluated after 2 min of growth factor stimulation, a time frame in accordance with the literature [26, 27]. The fact that we were unable to detect any difference in cdc42 activation between unstimulated, growth factor-stimulated and RV-treated cells fits to a recent study performed in colonic epithelial cells showing that cdc42 is dispensable for EGF-induced migration [15]. Moreover, the requirement of cdc42 for filopodia formation seems to be cell-type dependent and fibroblastoid cells seem not to be reliant on cdc42 [25].

Taken together, we could highlight a novel stimulus-specific anti-migratory action of RV, which is most likely the result of impaired Rac1 activation and subsequently reduced lamellipodia formation. Moreover, this study is the first to discover an anti-migratory potential of RV in classical growth factor-activated VSMCs.

## Abbreviations

CS: calf serum
DMEM: Dulbecco's Modified Eagles Medium
EGF: epidermal growth factor
PDGF: plateletderived growth factor
PI3K: phosphatidylinositol-3 kinase
RV: resveratrol
VSMC: vascular smooth muscle cell
