# Supplementary material for: Resveratrol inhibits migration and Rac1 activation in EGF- but not PDGF-activated vascular smooth muscle cells
Source: Mol Nutr Food Res. 2011 Jul 5;55(8):1230–6. doi: 10.1002/mnfr.201100309 (PMC3482936; doi:10.1002/mnfr.201100309)
Supplement: Supplementary file 1 [file mnfr0055-1230-SD1.pdf]

Supplemental Material

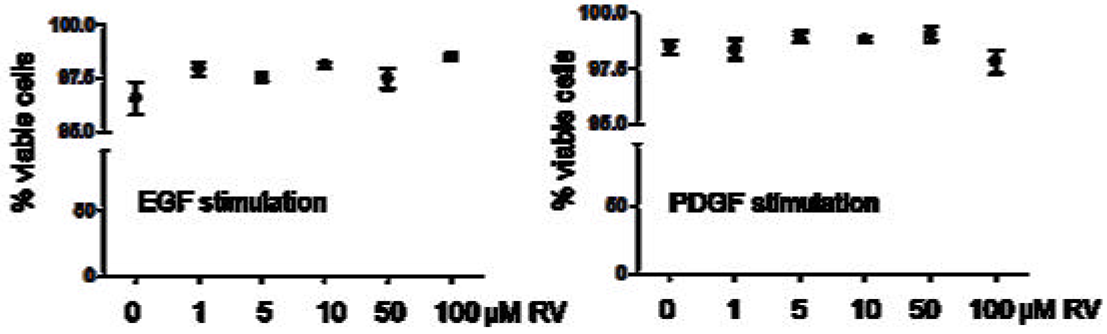

Supplemental Figure 1: RV does not reduce cell viability of rat VSMC up to a concentration of 100 µM.

VSMC were starved, treated with different concentrations of RV for 30 min and then stimulated with EGF (20 ng/mL) or PDGF (2 ng/mL) for 21 h, as done for the migration assays. Cells were trypsinized and viability was automatically assessed via Trypan Blue exclusion in a ViCell Analyzer (Beckman Coulter). The graphs depict compiled mean ± SEM of the percentage of viable cells out of three independent experiments.

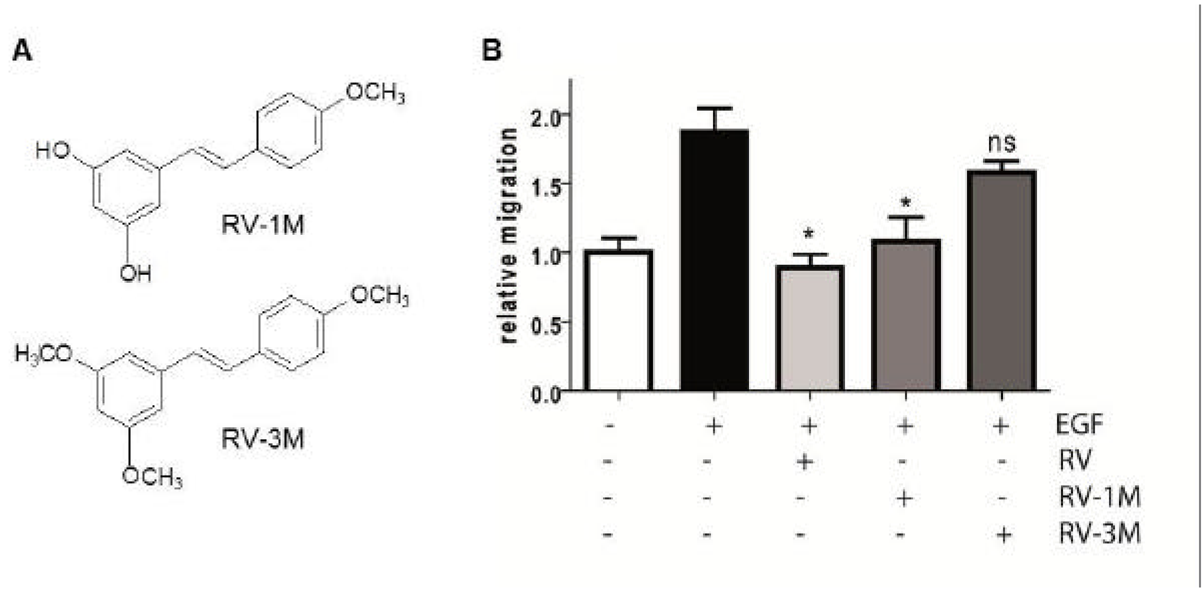

Supplemental Figure 2: RV inhibits EGF-induced VSMC migration in an apparently redox-independent manner.

(A) Non-antioxidant RV-derivatives of RV: *trans*-3,5-dihydroxy-4´-methoxystilbene (RV-1M) and *trans*-3,5,4´-trimethoxystilbene (RV-3M) (for extensive confirmation of their redox-inactivity please refer to [1])

(B) Starved confluent VSMC were preincubated with DMSO, 50 µM of RV or respective RV-derivative for 30 min prior to addition of EGF (20 ng/mL) for 21 h. Migration was assessed and quantified as described in the *Materials and Methods* section of the main manuscript. Bar graphs represent fold migration of unstimulated control and depict mean ± SEM of four independent experiments (*, $p < 0.05$; ANOVA, Dunnett´s post test vs. stimulated control (black bar))

✍ **Both RV and its knowingly non-antioxidant 4'-methoxy-derivative significantly inhibit EGF-induced VSMC migration in contrast to the tri-methoxylated derivative. This indicates that the antimigratory property of RV does not rely on its redox-activity, but rather on the presence of the hydroxyl groups in 3- and 5- position that might be necessary for selective molecular targeting.**

1. Schreiner, C.E., Kumerz, M., Gesslbauer, J., Schachner D., Joa, H., Erker, T., Heiss, E.H., Dirsch, V.M., Resveratrol blocks Akt activation in angiotensin II- or EGF-stimulated vascuklar smooth muscle cells in a redox-independent manner. *Cardiovasc. Res*. 2011, 90, 140-147.
